# Varicose Vein Education and Informed coNsent (VVEIN) study: a randomised controlled pilot feasibility study

**DOI:** 10.1186/s40814-023-01336-9

**Published:** 2023-06-22

**Authors:** Aoife Kiernan, Fiona Boland, Daragh Moneley, Frank Doyle, Denis W. Harkin

**Affiliations:** 1 Strategic Academic Research (StAR) Programme, Royal College of Surgeons, Dublin, Ireland; 2 Department of Vascular Surgery, Bon Secours Health System, Dublin, Ireland; 3 Data Science Centre, School of Population Health, RCSI University of Medicine and Health Sciences, Dublin, Ireland; 4 Department of Vascular Surgery, Beaumont Hospital, Dublin, Ireland; 5 Department of Health Psychology, School of Population Health, RCSI University of Medicine and Health Sciences, Dublin, Ireland; 6 Department of Medical Professionalism, RCSI University of Medicine and Health Sciences, Dublin, Ireland

**Keywords:** Consent, Digital consent, Health education tool, Endovenous thermal ablation, Vascular surgery, Venous disease

## Abstract

**Introduction:**

Doctors have a legal requirement and duty of care to ensure patients are enabled to make an informed decision about their treatment, including discussion of the benefits, risks and alternatives to a procedure. A patient-centred approach to consent has been firmly established in Ireland, and fundamental to this is the ability to engage in a dialogue that offers comprehensible information to patients. Telemedicine has revolutionised the way we can deliver care to patients in the modern era of computers, tablets, and smartphones, and its use has been rapidly expanded. Novel digital strategies to improve the informed consent process for surgical procedures have been increasingly under investigation over the last 10–15 years and may offer a low cost, accessible and tailored solution to consent for surgical interventions. Within vascular surgery, superficial venous interventions have been associated with a high number medicolegal claims and also represents an area within the specialty with rapidly evolving technology and techniques. The ability to communicate comprehensible information to patients has never been greater. Thus, the author’s aim is to explore whether it is feasible and acceptable to deliver a digital health education intervention to patients undergoing endovenous thermal ablation (EVTA) to supplement the consent process.

**Methods:**

This is a prospective, single centre, randomised controlled, feasibility trial recruiting patients with chronic venous disease deemed suitable to undergo EVTA. Patients will be randomised to receive either standard consent (SC) or a newly developed digital health education tool (dHET). The primary outcome is feasibility; assessing the recruitment and retention rate of participants and assessing acceptability of the intervention. Secondary outcomes include knowledge retention, anxiety and satisfaction. This feasibility trial is designed to recruit 40 patients, which will allow for a moderate dropout rate. This pilot study will inform the authors of the appropriateness of an adequately powered multicentre trial.

**Discussion:**

To examine the role of a digital consent solution for EVTA. This may improve and standardise the consent dialogue with patients and may have the potential to reduce claims related to poor consent processes and disclosure of risks.

**Ethical committee reference:**

Ethical approval has been sought and received from both the Bon Secours Hospital and RCSI (202109017), on 14 May 2021 and 10 October 2021, respectively.

**Trial registration:**

ClinicalTrials.gov Identifier: NCT05261412, registered on 1 March 2022

**Supplementary Information:**

The online version contains supplementary material available at 10.1186/s40814-023-01336-9.

## Introduction

### Background

Varicose veins (VV) affect one third of the adult population, and chronic venous disease (CVD) has a negative effect on quality of life (QoL), which can be significantly improved by treatment [1–4]. Chronic venous insufficiency (CVI) can be complicated by venous ulceration in over 3% of patients, and chronic treatment with dressings has been estimated to consume 2% of the health budget. Over the last 15 years, minimally invasive endovenous techniques to treat VV have been introduced and are proven to be cost-effective and safe, particularly when performed under a local anaesthetic in an outpatient setting [5]. The American Venous Forum, in 2011, and the National Institute for Health and Care Excellence (NICE), in 2013, have recommended endovenous thermal ablation techniques (EVTA), as the first-line treatments for truncal venous reflux [6, 7].

Doctors have a legal requirement and duty of care to ensure patients are enabled to make an informed decision about their treatment, including discussion of the benefits, risks and alternatives. This is reflected in the Health Service Executive (HSE) National Consent Policy and Irish Medical Council ‘Guide to Professional Conduct and Ethics’ [8]. A patient-centred approach to consent has been firmly established in Ireland since 2000 and reminds us that we have a duty to involve patients in decisions about their treatment of care and to engage in a dialogue that offers comprehensible information. This is in keeping with the fundamental ethical principle of autonomy [8].

Interventions to improve information transfer and comprehension in the consent process, such as providing standard patient information leaflets (PIL), report mixed results [9]. Information leaflets used during the process of consent have been shown to increase patient factual recall and satisfaction with the consent process and are considered best practice [10–16]. However, even well considered PILs, co-designed with patient or client engagement, do not always cover the less common areas of concern or risk which may be material to an individual patient [9]. More rigorous approaches are time and cost intensive, and can adversely impact on the efficiency of healthcare delivery, which limits scalability.

Telemedicine has revolutionised the way we can deliver care to patients in the modern era of computers, tablets and smartphones, and its use has been rapidly expanded [15]. Digital platforms are a novel tool to potentially improve dialogue and communication between doctors and patients. Patients in general have high satisfaction ratings for telemedicine, but certain patient groups may be less likely to engage or benefit from it on account of disability, technological illiteracy or access [15]. Therefore, the use of novel digital technologies for consent may offer a low cost, accessible and tailored solution.

### Rationale for study

Providing patients with understandable information is essential in order to respect and promote patients’ autonomy and protect them from harm. In healthcare settings, informed consent has a specific function to provide an instrument to guarantee a balanced doctor-patient relationship, whereby the patient gives explicit authorisation to accept or refuse treatment offered by the doctor. Novel digital strategies to improve the informed consent process (disclosure of information and its comprehension) for surgical procedures have been increasingly under investigation over the last 10–15 years [17–19]. Recently, a study by Gesualdo et al. reported that digital technologies for informed consent do not negatively affect patients and appear desirable in a surgical setting [20].

Vascular surgery is underrepresented in the aforementioned studies of novel strategies for informed consent, with only one study by Bowers et al. which included four minimally invasive vascular procedures but did not include EVTA [21]. Previous studies have reported that VV procedures were the primary source for medico-legal claims in vascular surgery [22–24] and were involved in 48% of successful claims in one study from the United Kingdom (UK) [24]. Although these studies likely represented mostly open VV surgery, vascular interventions are constantly evolving to become less invasive as technology advances and thus the ability to disclose comprehensible information to patients has never been greater. Improved consent and better communication with patients could significantly reduce the number of future claims. Pre-prepared consent forms to promote standardisation of the consent process could also potentially reduce claims [25].

Thus, we aim to explore whether it is feasible and acceptable to deliver a digital health education intervention to patients undergoing EVTA to supplement the consent process. The rationale for this study is to assess whether conducting a large definitive randomised control trial with this protocol is feasible in a busy day-surgery practice.

## Aims and objectives

### Aim

The authors’ aim to undertake a methodologically robust, single-centre, randomised controlled pilot feasibility trial assessing the feasibility of introducing a digital health education tool (dHET) for VV consent into a busy surgical practice. Our secondary aim is to explore whether the intervention has any impact on patients’ knowledge-recall, satisfaction or anxiety.

### Primary objective

The primary objective is as follows: to determine if the introduction of a digital health education tool for consent is feasible and practical in a busy day surgery practice by examining the number of eligible participants, recruitment rates to the study, retention rates of participants, acceptability of the study, protocol adherence, barriers to delivery of the assigned interventions and the time taken to complete the assigned intervention.

### Secondary objectives

The VVEIN pilot trial also includes secondary objectives related to the potential effect of the digital health education tool on knowledge recall, satisfaction and anxiety. Secondary objectives will be determined by examining the effect of dHET on early knowledge recall, patient satisfaction with the consent process, delayed knowledge recall, anxiety and satisfaction (at 2 weeks), the time spent by patients with the responsible surgeon and the number of questions asked by patients before signing consent.

### PICO

#### Population

The population includes patients with superficial venous incompetence undergoing an EVTA procedure.

#### Intervention

The intervention is as follows: digital health education tool (dHET) (multimedia file with video animation, narration and graphics) for informed consent for EVTA procedures—developed with EIDO^TM^healthcare and delivered on a handheld tablet device.

#### Comparison

Standard consent (verbal discussion + written information leaflet EIDO™ healthcare)

Primary outcomes:

- Eligible participants
- Recruitment number
- Retention number
- Acceptability
- Adherence to protocol
- Barriers to assigned intervention
- Time taken to complete assigned intervention

Secondary outcomes:

- Early knowledge recall
- Patient satisfaction
- Patient anxiety
- Delayed knowledge recall
- Time spent with surgeon
- Number of questions asked by patient

## Study methods

### Statement of design

The VVEIN study is a two-arm, randomised pilot feasibility trial to assess the practicality of supplementing the consent process for EVTA procedures with a dHET. This randomised feasibility study shall be carried out in accordance with the guidance set out by the Consolidated Standards of Reporting Trials (CONSORT) group for randomised pilot and feasibility trials. http://www.consort-statement.org [26]. The VVEIN study has been prospectively registered on ClinicalTrials.gov. (NCT Identifier: NCT05261412).

**Fig. 1:**
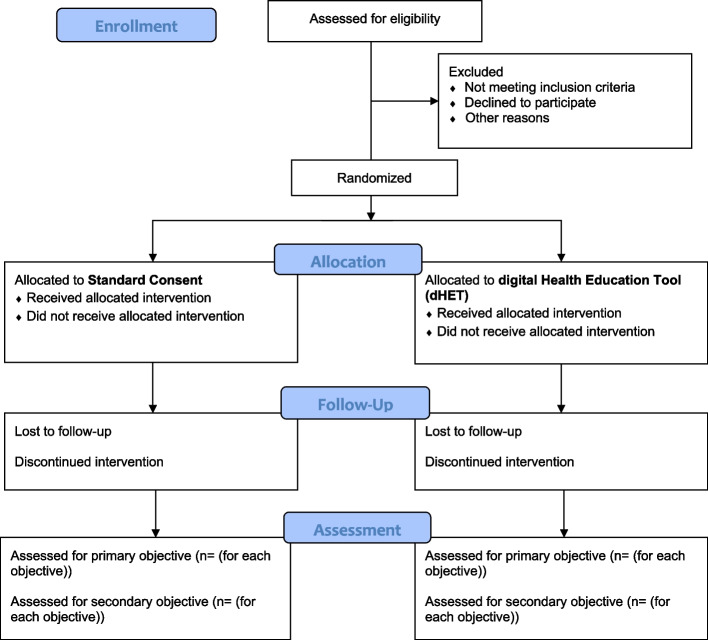
CONSORT flow diagram for VVEIN pilot feasibility study

Participants shall be randomised in a 1:1 ratio to one of two parallel groups. Study flow is shown in Fig. 1. The dHET was co-designed with EIDO healthcare and contains information about EVTA procedures and the benefits, alternatives and risks of the procedure. EIDO healthcare information is delivered in a multimedia file with video animation, narration and graphics.

### Study flow

The study flow is shown in Fig. 1.

### Participants

For the purpose of this pilot study, two vascular surgeons at the study location will be recruited to participate in the trial. Two were selected for pragmatic reasons in this initial pilot study. All consenting patients attending the vascular outpatient department of the two recruited surgeons, with truncal saphenous vein incompetence, suitable for an EVTA procedure (with or without adjunctive procedures (phlebectomy/foam sclerotherapy)) will be assessed for eligibility. Where both legs are being treated, only the first leg/episode of consent shall be randomised to the study.

#### Inclusion criteria

- Deemed suitable for EVTA by treating surgeon
- First procedure for superficial venous incompetence
- Full consent
-  > 18 years
- Proficient in English

#### Exclusion criteria

- Redo or second procedure for superficial venous incompetence (in same or opposite leg)
- Cognitive impairment or unable to consent
- Not meeting inclusion criteria

### Expected study duration

Ethical approval was sought and approved in May 2021 from the research ethics committees (REC) at the study location and at the Royal College of Surgeons in Ireland (RCSI). Recruitment will commence in Spring 2022 and continue over a maximum 12-month period until March 2023 or until recruitment targets are met. Subsequent amalgamation of data and analysis will be performed upon completion with the final results expected thereafter.

### Study setting

Patients who meet the inclusion criteria will be identified at the surgical outpatient department at the study location and invited to participate by a member of the research team. An initial consent and information process shall be carried out with patients deemed suitable for EVTA by their responsible surgeon. All patients (agreeable to participate and those unsure) will be provided with a PIL for the study approved by the local REC, for education purposes. Upon re-presentation to the day ward for their procedure, patients will be verbally reconsented for inclusion prior to randomisation and allocation to a study arm.

### Intervention

- *Control group*: Participants in the control arm will undergo *Standard Consent (SC)* which will consist of paper PIL provided by EIDO™ healthcare followed by a verbal discussion (standardised by following a checklist of topics to discuss) with the responsible consultant surgeon and signing of their consent form. The time taken to read the PIL (recorded with a stopwatch) will be recorded. The time spent with the responsible surgeon will also be recorded, as will the number of questions asked by the patient.
- *Intervention*: Participants randomised to the intervention dHET will receive the dHET followed by a verbal discussion (as above) and signing of their consent form. The dHET will be delivered on a tablet computer and facilitated by a research assistant who will ensure all technological issues are overcome but will not engage with the participant with regard facilitating better understanding of the content. The digital offering will be interactive; patients will be able to traverse through each section at their own pace with the ability to re-visit sections as many times as they wish. It also contains a short narrated animation of the procedure, which they can play, rewind or fast forward. The time spent reading each section of the dHET and time spent watching the animation will be recorded. The time spent with the responsible surgeon will also be recorded, as will the number of questions asked by the patient.

All patients will complete a knowledge questionnaire at baseline, post intervention on the day of surgery and at the 2-week follow-up telephone interview. As no validated knowledge questionnaire for EVTA procedures was available, we developed one based on information provided in the EIDO^TM^ PIL and from a separate body of work by our team—expert consensus for essential information for varicose vein surgery—a modified Delphi study [27]. The knowledge questionnaire consists of 20 True or False questions. Patients are encouraged not to guess and to choose ‘Unsure’ if they do not know the answer. The questionnaire was piloted among post-operative EVTA patients and a mean score of 10.39 was achieved.

The six-item State trait Anxiety Inventory (STAI-6) will also be completed at baseline, post intervention on the day of surgery and at the 2-week follow-up telephone interview. The short form of the STAI was developed for use in circumstances when the full form is inappropriate, such as a busy day-surgery ward. It correlates closely with the full-form and has acceptable reliability and validity [28].

The client satisfaction questionnaire (CSQ-8) will be administered on the day of surgery and repeated at the 2-week follow-up telephone interview. The CSQ-8 questionnaire consists of eight self-report questions, constructed with a four-point Likert scale reply. The minimum achievable score is 8, indicating poor satisfaction, and maximum score is 32, indicating a high level of satisfaction. This tool has been found to be acceptable in studies examining patient satisfaction with consenting methods [29–32].

#### Contraindications, cautions and interactions to be considered

There are no contraindications or cautions to be considered when utilising this dHET to supplement the consent process. The information contained in the dHET reflects the HSE National Consent Policy and Irish Medical Council ‘Guide to Professionalism and Ethics’ [8]. No variation in risk to standard consent protocols has been identified.

### Data collection

Baseline patient demographic data will be collected pseudonymously from patients and/or from patient medical records. The following will be recorded:

- Age
- Gender
- Level of education (highest achieved)
  - Primary school
  - Secondary school
  - Third level
- Health literacy—rapid estimate of adult literacy in medicine (revised) REALM-R—score of 6 or less considered to be at risk for poor health literacy [33, 34]

Outcome data will be collected prospectively. Outcome data and definitions are provided below:

Primary outcomes

- ▪ Eligible participants
  Number of eligible participants (meeting inclusion criteria)
- ▪ Recruitment number
  Number of participants consenting to participate
- ▪ Retention rate
  (Number of patients who consent to participation) minus (number of patients who voluntarily withdraw) divided by (number of subjects who were randomised)
• Acceptability
  Measure of the perception among study personnel and patients that the dHET is agreeable or satisfactory. Measured on a 5-point Likert scale for study personnel (where 5 is highly acceptable and 1 is poorly acceptable) and using the post intervention CSQ-8 survey for patients.
- ▪ Adherence to protocol
  Number of patients who completed their assigned intervention, also includes the proportion of complete data for each outcome measure (CSQ-8, STAI-6, REALM-R, demographic data collection)
- ▪ Barriers to assigned intervention
  Number of patients not randomised due to staffing or time constraints (with reason recorded), technology issues with tablet/dHET/link to knowledge quiz/internet access
• Time
  Time (minutes) taken to complete the assigned intervention (and any delays caused as a result)

Secondary outcomes:

- Knowledge recall
  Number of answers correct in True/False/Unsure questionnaire, where a correct answer = 1 and incorrect/unsure = 0 (max score 20).
- Patient satisfaction
  Measured using Client Satisfaction Questionnaire (CSQ-8) on the day of surgery and at 2-week follow-up phone call, a validated questionnaire to assess consumer satisfaction with health services. Scores range from 8 to 32, with higher values indicating higher satisfaction [30, 32, 33, 35].
- Patient anxiety
  Measured at baseline, after the intervention and at the 2-week follow-up phone call, using the six-item State-Trait Anxiety Inventory (STAI-6), a short-form version of the state scale, consisting of six items chosen for reliability and validity, which produces scores that are comparable to using the full version [28].
- Delayed knowledge recall (at 2-week follow-up)
  Number of answers correct in True/False/Unsure questionnaire, where a correct answer = 1 and incorrect/unsure = 0 (max score 20).
- Time spent with surgeon
  Time (minutes) spent with surgeon
- Number of questions asked by patients
  Number of questions patient asks after assigned intervention but before signing consent form

### Sample size

As this is a pilot study, a formal sample size calculation was not performed [36]. Sample sizes of between 24 (12 per group) and 50 have been recommended variously for pilot studies [37–40]. Following these broad recommendations, we chose a recruitment sample size of 40 (20 per group) which would allow for a moderate dropout rate. A significant dropout rate (e.g. 40%) would reduce the pilot sample size to below a minimum 24, in which case a planned larger study would be called into question in the first place, having possible external validity issues, pragmatic or ethical concerns.

### Interim analysis and stopping guidelines

As this is a pilot study run over a short time frame, we do not envisage a scenario where the trial will be ceased early.

### Randomisation

- Sequence generation: generation of a random sequence will be performed using a computer-based programme by the trial statistician who will not have any contact with trial participants.
- Randomisation type: block randomisation (in blocks of two, four, six)
- Allocation concealment: assignments shall be enclosed in sequentially numbered, opaque, sealed envelopes and stored securely in a locked filing cabinet.
- Implementation: the trial statistician will generate the allocation sequence. They shall not have any direct contact with the study participants. Recruitment shall be carried out by the surgical team (consultant/research team). Upon confirmation of consent on the day of surgery, a numbered envelope containing randomisation data will be selected in sequence and the allocation assigned to the patient. The research assistant will be blind to the allocation sequence only. A unique study number will be assigned to each individual at the time of randomisation. All data collected will be input electronically into a database by the research assistant for analysis upon completion of the study.

### Blinding

By the nature of the intervention the participating patients will not be blinded. The responsible surgeon confirming consent prior to the procedure will be blind to participant allocation. The research assistant will be blind to the allocation sequence until opening of the sealed opaque envelope.

### Ethical approval and data protection

Ethical approval has been sought and received from both the Bon Secours Hospital and RCSI (202109017), on 14 May 2021 and 10 October 2021, respectively. All data will be pseudonymised (study number) and stored securely in a password-protected file in the Bon Secours Hospital systems for the duration of the study. Once analysis has been completed, data will be irrevocably anonymised by destroying the master key.

## Trial progression

### Continuation criteria

Continuation criteria (Table 1) will be considered to determine whether further evaluation of this intervention is warranted (to test the effectiveness of the intervention) [41, 42]. These criteria are primarily based around the primary objective of feasibility and the potential for effectiveness and system-wide implementation.

**Table 1:** Continuation criteria VVEIN pilot study

	**Proceed to RCT**	**Proceed to RCT following some changes to the protocol**	**Do not proceed to RCT unless problems can be overcome**
Recruitment	Recruitment of ≥ 80% study participants within 4 months	Recruitment of ≥ 60% study participants within 4 months [41]	Recruitment of < 60% study participants within 4 months
Retention	Retention of ≥ 80% of study participants for follow-up	Retention of ≥ 60% of study participants for follow-up	Retention of < 60% of study participants for follow-up
Feasibility and acceptability	dHET intervention on day ward acceptable to > 75% of study personnel (score of 4 or 5 on Likert scale) and patients involved (score of 28 or above on CSQ-8)	dHET intervention on day ward acceptable to > 50–74% of study personnel (score of 4 or 5 on Likert scale) and patients involved (score of 28 or above on CSQ-8)	dHET intervention on day ward acceptable to < 50% of study personnel (score of 4 or 5 on Likert scale) and patients involved (score of 28 or above on CSQ-8)
Non-adherence	Non-adherence to the intervention in < 10% (dHET and/or DOS questionnaires incomplete)	Non-adherence to the intervention in > 11–20% (dHET and/or DOS questionnaires incomplete)	Non-adherence to the intervention in > 21% (dHET and/or DOS questionnaires incomplete)

*dHET* Digital Health Education Tool, *DOS* Day of surgery

### Subject withdrawal

Any participant can withdraw consent for participation at any point; they do no need to give a reason. Any participant wishing to withdraw can do so verbally or in writing to a member of the research team.

The investigator may withdraw a patient from the study if they:

- Experience a serious or intolerable adverse event
- Develops, during the course of the study, a condition which renders the patient unfit for intervention
- Requires early discontinuation for any reason

### Adverse events

EVTA is considered a very safe ambulatory vein treatment, and risk of adverse events would be considered very low [4, 6, 43–45]. An adverse event is defined as any undesirable medical experience by a patient, whether or not it is related to the intervention. A severe adverse event (SAE) is a life-threatening experience. All adverse events in a patient enrolled in this study, regardless of its causal relationship to study treatment, will be recorded and shall be documented in the final report. The principal investigator will be notified of all SAEs within 24 h. All SAEs will be reported according to the policies of the institution and the approving research ethics committee.

### Follow-up

Participants shall be contacted by one researcher via telephone at two weeks for a virtual appointment and to complete the delayed knowledge recall questionnaire. Patients will also be followed up in a standardised fashion at 6 weeks in the vascular outpatient department at the study location or sooner should the need arise.

### Trial closure

The trial shall be considered completed when a total of 40 participants have been randomised, as this will allow for up to 20% dropout rate.

## Confidentiality statement

All patient data will be stored securely in a password-protected database on the Bon Secours systems under the guidelines of the Data Protection Act and the EU General Data Protection Regulations (GDPR) 2016. Patient details will be pseudonymised as each participant will be allocated a study number. The codes for the allocated study numbers will be kept in a secure password-protected file, only accessible by the principal investigator and trial lead. The principal investigator will preserve the confidentiality of the participants taking part in the study and is registered under the Data Protection Act.

Patient details, including contact information, recorded on paper forms, including the questionnaires and consent forms will be scanned or data entered manually to the database described above. In line with RCSI policy, data will be kept for research purposes for a total of five years, after which the files will be destroyed.

## Discussion

The standard of care and ethical obligation of physicians’ is to ensure a patient is adequately informed prior to consenting to any invasive procedure. For many physicians working in a busy surgical practice such as vascular surgery, the process of informed consent has become increasingly difficult due to increased patients’ expectations and evolving technology within the speciality that can increase complexity of the procedures [21]. Several studies have shown benefit of digital tools, especially with active patient participation for improving patients’ understanding and satisfaction of informed consent [46, 47], but vascular surgery is underrepresented in these. In the context of a fast-paced surgical day ward where patient throughput is high and only minor delays in processes are accepted to ensure efficiency, this pilot study will support a decision to scale-up to a multicentre randomised controlled trial (RCT) if recruitment and retention is adequate.

The purpose of a future multi-centre RCT that could compare standard consent, which is the current practice, to a digital health education tool to assess the primary outcome measure of patients’ knowledge recall, would likely inform physician’s practices around informed consent and will be generalisable to other vascular and surgical procedures.

## Status of trial

Protocol: 1: V5

Recruitment: Spring 2022

Estimated completion: July 2023

## Supplementary Information

**Additional file 1: Figure 1.** SPIRIT figure.

## Data Availability

Anonymous data and materials will be encrypted and stored in the secure server within the Bon Secours Health system. Data will be available upon suitable request to the senior author.

## Abbreviations

CSQ-8: Client satisfaction questionnaire-8
CVI: Chronic venous insufficiency
dHET: Digital health education tool
DOS: Day of surgery
EVTA: Endovenous thermal ablation
GDPR: General data protection regulations
HSE: Health Service Executive
NICE: National Institute of Clinical Excellence
PIL: Patient information leaflet
QoL: Quality of life
REALM-R: Rapid estimate of adult literacy in medicine, revised
RCSI: Royal College of Surgeons in Ireland
RCT: Randomised controlled trial
REC: Research ethics committee
SAE: Severe adverse event
STAI-6: State trait anxiety inventory-6
UK: United Kingdom
VV: Varicose veins
