# Supplementary material for: Varicose Vein Education and Informed coNsent (VVEIN) study: a randomised controlled pilot feasibility study
Source: Pilot Feasibility Stud. 2023 Jun 22;9:104. doi: 10.1186/s40814-023-01336-9 (PMC10286428; doi:10.1186/s40814-023-01336-9)
Supplement: Supplementary file 1 — Additional file 1: Figure 1. SPIRIT figure. [file 40814_2023_1336_MOESM1_ESM.doc]

Figure 1. SPIRIT figure

|  | **STUDY PERIOD** | | | |
| --- | --- | --- | --- | --- |
|  | **Enrolment** | **Allocation** | **Post-allocation** | |
| **TIMEPOINT**** | ***-t_1_*** | **0** | ***Day of surgery*** | ***2 weeks*** |
| **ENROLMENT:** |  |  |  |  |
| **Eligibility screen** | X |  |  |  |
| **Informed consent** | X |  |  |  |
|  |  |  |  |  |
| **Allocation** |  | X |  |  |
| **INTERVENTIONS:** |  |  |  |  |
| ***[Standard Consent]*** |  |  | X | X |
| ***[Digital health education tool ]*** |  |  | X | X |
|  |  |  |  |  |
| **ASSESSMENTS:** |  |  |  |  |
| ***[Knowledge]*** | X |  | X | X |
| ***[Anxiety (STAI-6)]*** | X |  | X | X |
| ***[Satisfaction]*** |  |  | X | X |
| ***[Demographics]*** | X |  |  |  |
| ***[Level of education]*** | X |  |  |  |
| ***[Health Literacy]*** | X |  |  |  |

*Recommended content can be displayed using various schematic formats. See SPIRIT 2013 Explanation and Elaboration for examples from protocols.

**List specific timepoints in this row.
